# A Humane Social Learning-Informed Metaverse: Cultivating Positive Technology Experiences in Digital Learning Environments

**DOI:** 10.1089/cyber.2023.0001

**Published:** 2024-01-08

**Authors:** Andrew Villamil, Sará King

**Affiliations:** ^1^ Department of Behavioral and Organizational Sciences, Claremont Graduate University, Claremont, California, USA.; ^2^ Department of Public Health, University of California San Diego, La Jolla, California, USA.

**Keywords:** technology, metaverse, social learning theory, social media, artificial intelligence, adolescence, social media, environment, positive psychology, learning, engagement, digital learning

## Abstract

Online environments, such as metaverses, provide distinct social environments for people to engage in complex, cognitive, and multidirectional learning and meaning-making experiences. These engaging and influential environments highlight important factors associated with the Social Learning Theory (a process through which external settings influence behavior in specific environments). According to this theory, environments provide a space for youth to engage in reciprocal interactions of interpersonal, behavioral, and environmental cues. Online environments designed by social media companies have been scrutinized, given their dependence on algorithms (artificial intelligence systems). Research has revealed the effects of systems that use machine learning to subversively maintain engagement on their platforms for as long as possible. Given the constant changes in socializing environments, younger generational cohorts need to be adequately prepared for systems that determine what type of content they are exposed to, and shape the timing, frequency, and agentic influencers they engage with. Therefore, this article proposes a necessity to expand our understanding about social learning and current technology design principles. This article demonstrates the need for a paradigm shift toward exploring an innovative construct referred to as the digital learning environment. We examine existing issues in the design of digital spaces, provide a positive developmental psychology framework that informs further research, and propose solutions for researchers, educators, policymakers, and caregivers as they navigate healthy technology use and predominant mental health issues in the 21st century.

## Introduction

Immersive technologies such as extended reality (XR), virtual reality (VR), augmented reality (AR), mixed reality (MR), video games, and social networking sites have become modes through which individuals can connect, engage in experiences, and explore digital learning environments and spaces. For the purposes of this article, we define digital learning environments as any digital environment in which a person has an opportunity to experience or learn behaviors that impact how they make sense of and engage with the world. We propose the development of Digital Learning Theory as a means of understanding the complex interactions that are present within digital learning environments, including guiding or shaping the ways people learn to perceive their relationship with a constantly evolving sense of self.

Given the recent negative effects from technology design that prioritizes engagement at any cost, we posit that there is a need to integrate positive psychological theories with positive design principles. Incorporating dynamics from Bandura's Social Cognitive Theory, an advancement to traditional Social Learning Theory, we propose that Digital Learning Theory demonstrates opportunities for “reciprocal determinism,” in digital environments. Reciprocal determinism emphasizes that there is a constant interactive relationship between personal factors, behaviors, and environments where all elements are continuously influencing each other in unique and dynamic ways,^1^ although these elements play out specifically in the environment of any digital platform.

Digital learning environments provide an important distinction between technological and physical learning environments in that a person's experiences are always mediated by digital technology in a digital environment, whereas one can have experiences that are not mediated by digital technology in physical learning environments. These two environments can co-occur and mutually influence one another, such as using digital education platforms in a physical classroom environment. Digital learning environments can exist in different forms, with some of the most popular devices and platforms providing opportunities for both learning and interaction. Currently, some platforms and devices that facilitate digital learning environments include but are not limited to Oculus, Instagram, Xbox, PlayStation, Nintendo, Apple, Meta, Snapchat, Sandbox, Decentraland, Vatom, Roblox, and V.R. Chat, YouTube, TikTok, Threads, and X (formerly Twitter). Many of these platforms are being used individually or combined, over 3 hours per day by Generation Z.^2^

These platforms appear to present significant time and opportunity for digital agency. Users tend to believe they are the “curators” of the experiences they are exposed to due to the opportunities for human engagement through connecting with peers to comment, view, or like their posts. Whether they are interacting with peers, or searching for topics or groups in these digital landscapes, “human engagement” influences how individuals interact with digital platforms and each other, and the result is that many users believe that they are “self-selecting” or curating their experiences independently.^3–6^

Recent revelations from the technology industry have demonstrated that this is only occasionally the case.^3,7^ The introduction of content curation algorithms powered by artificial intelligence was a significant advancement in how technology can be used in digital landscapes. Algorithms introduce additional variables that may be positive (e.g., humor, awe-inspiring content) or negative (e.g., harmful content, hate speech).^4^ In addition, algorithms may introduce experiences in which cyberbullying or trolling (offensive messaging to sow discord) may occur. These types of harmful environments can influence negative behaviors. For example, an internal study by a major technology company has demonstrated that teenagers who suffer from negative technology experiences become accustomed to abusive patterns of behavior from other users (agentic operators or influencers), which distorts their ability to cognitively authenticate a sense of trust and safety in people in the physical world around them.^8,9^

Agentic operators or influencers, according to Albert Bandura, are individuals who actively engage in behaviors that modify the world around them (i.e., self, others, environment). This lack of trust negatively impacts self-confidence and increases feelings of isolation and depression.^4^ These experiences lead to behaviors that promote alienation and withdrawal, which then reinforce negative emotions such as depression and loneliness.^10^ Since individuals have relatively little control over what they are exposed to when engaging online, the revelation of these aforementioned adverse effects highlights the need to consider how digital landscapes facilitate learning for individuals in sensitive developmental periods (e.g., childhood, adolescence, young adulthood, and older adulthood). In addition, youth from marginalized backgrounds may be at risk for a greater prevalence of mental health disparities and adverse childhood experiences (ACES) due to systemic oppression.^11–13^

Consequently, this can impede their ability to access the kinds of community-based stress buffers that might otherwise shield them from negative social and emotional experiences, which can be further compounded by interactions in digital learning environments if these are not constructed with their needs and perspectives in mind.

Online interactions between peers and environments can be compared with the experiences humans encounter across their life span in equivalent physical spaces. Humans learn through observation and interaction.^5,14,15^ Social Learning Theory represents the processes and cognitive mechanisms of learning; specifically, that humans learn new behaviors through observation and interactions with their environments.^16^

Human experiences in environments cognitively mediate learning, and current research highlights how online environments are now also essential to learning in youth.^14,17–19^ Online environment experiences translate to learning effects in individuals, which, in turn, alter basic learning processes through engagement in digital and physical environments. We suggest that one's approach to the design of humane metaverses and other digital learning environments would be to consider the importance of human flourishing^20^ or with the components of awareness, insight, connection, and purpose in mind (discussed further in the Recommendations for Research and Design on Digital Learning Environments section) in order for designers to possibly enhance the well-being of people within those contexts. Designing with well-being in mind can be supported by applying developmental and positive psychological theories in designs that counteract potential negative technology experiences and enhance opportunities for optimal experiences, which in turn will elicit positive outcomes.

### Negative technology experiences

Much research has focused on the outcomes of negative technology use, including loneliness/disconnection,^21^ radicalization,^22,23^ anxiety, depression,^24^ anger, disinformation,^25^ and violence,^26,27^ among others. Most of these issues have been attributed to the targeted use of artificial intelligence designed to ensure that users spend as much time as possible in digital environments.^3,7,24,28^ Recent revelations by researchers, former executives, and whistleblowers regarding technology companies' efforts to create algorithms that modify the online environments people are exposed to (through artificial intelligence and machine learning) have highlighted the practical and dangerous activities of digital systems that are inherently designed to reduce agency and promote certain behaviors in humans.^3,27^

Algorithms create composites that contain information such as “likes,” “shares,” and “comments,” in addition to other interactions the algorithm believes will increase engagement and will produce the lengthiest time of users' attentional resources.^3,6,29^ “Engagement of users” is at the center of current technology and human experiences. Internal research released by a whistleblower from a major technology company demonstrates that such engagement experiences significantly alter what people see, whom they interact with, where they connect with others, the perception of their agency, and how they may experience powerful negative emotions such as anger, depression, isolation, radicalization, comparison, or anxiety.^7,27^ Recent studies have demonstrated that technology algorithms can be so effective and efficient that they will bombard users with extreme content every 38 seconds spent on the platform.^28^

Negative technology experiences may be a natural progression of the current interactions with digital platforms. The purpose of these platforms is generally to attract advertisers and increase engagement (more time and interactions spent online) through algorithms that are unintentionally effective at preying on human negativity bias 3.^29–31^ Negativity bias is a salient physiological and cognitive process that occurs when negative influences, which require more attentional resources, are prioritized over positive ones.^32–34^ This bias is highly correlated with increased technology engagement.^31,35–37^ The propensity to engage in negative experiences can significantly distort an individual's perception of his or her agency and autonomy and lead to negative downstream consequences, including distrust of information and experiences shared with others.

Therefore, understanding the effects of algorithms and developing protocols to protect human agency with technology are an essential component for designing humane digital learning environments.^38^ One critical lens for understanding algorithms is approaching them through the lens of Social Learning Theory. Through this lens, algorithms serve as agentic influencers. Agentic influencers are objects or people whose actions shape each other's environments.^16^ Algorithms can shape cognitive perspectives, such as perceptions of freedom and self-influence, through efficient data processing, which can influence human behaviors.^2,39,40^

### Positive technology experiences through design

Given the intensity of the negativity bias, previous research has demonstrated that it is essential to create more positive experiences than negative ones (positivity ratio) to counter the attentional resources involved in negative emotions.^41^ When considering the current unprecedented mental health crisis among adolescents, as well as the global epidemic of loneliness and historically high rates of suicidality,^42^ it is essential to understand that any experience or environment that is contributing to an increase in negative emotions might have a harmful effect on our collective health and well-being. When technology is designed or used to promote positive healthy psychological experiences, there can be significant collective benefits. Human engagement in positive experiences can increase positive upward spirals,^43^ contributing to their overall sense of well-being. A positive upward spiral is defined as a process in which a positive event or influence leads to a chain of positive outcomes that reinforce each other.^44^

Therefore, a humane metaverse is best designed to promote abundant positive experiences. In this article, positive technology experiences are defined as experiences that elicit positive outcomes. For individuals to share positive technology experiences, digital learning environments should support agency and positive developmental opportunities informed by social, developmental, and positive psychology constructs. These areas represent some of the most critical dynamics in human interaction and learning. People who engage in positive technology experiences tend to report increased positive emotions,^39^ increased connections,^20,45^ positive perceptions of themselves and others, increased agency,^46^ increased prosocial behaviors,^47,48^ and report higher levels of cognitive performance.^49^ These individuals report less distrust^50^ and use technology in ways that are generative and demonstrate healthier engagement with their devices.^37,39,51,52^

We can therefore posit that for people who experience increased positive technology experiences at greater frequency than negative experiences, this might result reciprocally in increased positive emotional experiences of connecting to self and others. It would be interesting for future research to study whether positive technology experiences correlate with positive emotional experiences within the context of interpersonal relationships or community settings within nondigital environments, thus contributing to our overall understanding of the affective impact of digital learning environments.

Metaverses are digital learning environments that depend on user engagement. This engagement, however, is a delicate balance between positive and negative experiences, which, in turn, affects how people feel when they spend time in an environment.^38,41^ Therefore, it is helpful to consider these environments as active environments that will influence what people are exposed to and how they can engage in these environments in ways that lead to better outcomes.^53^

## Social Learning Theory

Researchers seeking to understand human behavior and development in the early 20th century sought to understand human changes through their “overt behavior.”^54^ Specifically, John Watson attributed learning through two traditional types: Classical Conditioning (connection between stimulus and response or reflex) and Operant Conditioning (deployment of rewards and punishments for behavior).^55^ Behavior modification is a method of gradual change implemented over time and through specific social interactions to reinforce “desirable behavior,”^51^ determined by another individual (e.g., caregiver, teacher). Skinner proposed a mechanism that is at the root of many social media algorithms, the construct of intermittent variable rewards, which is a mechanism that heavily influences human behaviors and has been used for decades, most famously by casinos and games.^3^ Intermittent variable rewards are a form of reinforcement in which rewards are provided at unpredictable intervals and are often used as a means of shaping and maintaining behaviors while increasing engagement.^56–58^

These explanations of human change over time provide the foundation for social learning theorists to explore other human behavior, personality, and cognitive changes in human beings in adolescence and across their life span.

The primary belief in Social Learning Theory is that personality can be learned from a young age and across the life span. Specifically, socialization is the process through which society trains youth to behave similar to “ideal adults of that society” within environments, usually through overt cues. This is important because Albert Bandura and Walters later introduced the concept of modeling and vicarious reinforcement, that is, the introduction of complex behaviors youth experience by observing others, which over time are combined and “cognitively organized” to create more complex behaviors.^13,59,60^ This cognitive organization, in turn, creates agency, as Bandura notes, when an individual intentionally influences his or her function and contributes to life circumstances.^38^ For cognitive organization to occur, individuals must interact with other people or agents in specific environments embedded within pervasive cultural belief systems.^51^ They must also be self-regulators and self-examiners of their functions.

In addition, as Bandura notes, human functioning is a “product of the reciprocal interplay of intrapersonal, behavioral, and environmental determinants.”^1^

Social Learning Theory provides an extensive foundation for terminology and understanding of human behavior because it highlights a series of complex cognitive and physical interactions that shape human development. The most significant elements in this article are the influences on the behaviors of individuals and the constructs of the environment-person-behavior system. This system involves three interdependent and important elements that influence human development. The first is the biological and psychological characteristics of the person; the second is the person's behavior (i.e., external behaviors); and the third is the environment (i.e., what the person is being exposed to and where they spend time). All three are essential in the interaction and development of the individual and influence each other, particularly during important periods of development. This knowledge paves the way for understanding how technology introduces unique environments for this triadic relationship to influence human behavior.

### Applying social learning theory in digital learning environments

Technology, specifically digital learning environments, such as social media sites, video games, and metaverses, provide distinct environments for people to engage in complex, cognitive, and physical behaviors.^8,15,61–63^ These online environments are influential when reviewing socialization through the three types of environments that Bandura refers to as “Model Environments.” The first model is the imposed environment, which is usually forced upon individuals that they cannot necessarily control (e.g., school). The second model environment is the selected environment; this is the part of the environment that people choose to engage with and select their interactions (e.g., specific courses, interest groups, metaverses, and other digital learning environments). The third model environment is the created environment; these are environments that people construct through behaviors (e.g., temperament, external behaviors).

It is important to note here that there can be overlap between the three model environments, whether they are situated in physical or digital learning environments. For instance, a person could be attending a school environment where he or she is offered the chance to choose between different digital classroom environments, and part of this class could be the opportunity to create his or her own metaverse or other digital learning environments in which he or she could invite others to explore.

These components, when experienced in development and cognitively processed, lead to what Bandura emphasized as “self-efficacy,” the perception of competence when interacting with one's environment and experiencing agency or influence over actions in one's life, specifically as it relates to teenagers and young adults as interpersonal relationships, physical appearance change, and other responsibilities are introduced. These experiences can be encountered at various times and places, and the environments in which individuals live can significantly alter the trajectory of development.^10,59,64^ Complex environments in digital landscapes may present abstract models that use covert techniques that influence proactive agency and alter observational learning.

Researchers have demonstrated that the Internet is a highly social environment for learning and human interaction.^44,58,65,66^ Given the nature of highly peer-promoted and versatile digital learning environments cultivated by social media platforms such as Instagram or Facebook, we can see that the term “environments” previously adopted by social learning theorists can apply to numerous circumstances and environments where adolescents frequently spend time and interact.

## Humane Development of a Metaverse

“Whether as “forum” or “frontier,” cyberspace's distant land is dangerous yet attractive, and invites “colonization.”^8^ As evidenced by Bandura, environments provide a crucial space for humans to explore the cognitive “maps” of their development. The complex interactions between biological characteristics, behavior, and the environment are sensitive across human development. While Bandura's theory is predominantly focused on physical environments, current day cognitive organization, resulting from complex human interactions, has been increasingly occurring through online interactions, especially among people in the 21st century.

When viewed as interactive tools, digital learning environments create opportunities for humane conditions between people and computers to learn from interactions. These tools demonstrate that online environments are potent agents of socialization, where learning and processing can occur in settings outside of the physical environments.^14,15,58^ Online platforms have altered our understanding of “learning culture”^60,62^ and created opportunities for intrapersonal experiences that influence cognition. Technology use has demonstrated that people are active and engaging agents of information and interactions crucial to creating and maintaining self-efficacy, sharing, discussing, and influencing each other by participating in the learning process through exploration and engagement.^48,50,56^ Using Social Learning Theory in positive ways, such as in shaping authentic and positive interactions or learning situations that account for inclusivity and the reduction of harmful content, can help provide safe challenges and positive outcomes.

## Recommendations for Research and Design on Digital Learning Environments

Technology provides several modes of interaction and engagement, and research and design in the field should draw from knowledge from several disciplines, including psychology, anthropology (due to the intersection of culture with interpretations and use of technology), human-centered design, and human–computer interaction,^67^ among others. Given that human development is a complex system of relationships across human interactions, it is important to understand how these interactions can be shaped with the aforementioned digital learning principles.^64^ Researchers have argued that one of the main challenges concerning technology implementation is creating more defined and common frameworks to support design as therapeutic interactions mediated through technology.^65^ In this section, we provide recommendations based on existing research that has been investigated relating to technology with recommendations that could also benefit from more comprehensive and transdisciplinary studies given the complex nature of technology.

Table 1 presents a list summarizing relevant supplementary concepts and additional elements that have been researched. This is not intended to be an exhaustive list of recommendations in a prescriptive sense; rather, these recommendations are meant to catalyze and promote further conversations and explore areas of inquiry among researchers, educators, policymakers, technology designers, caregivers, end users, and other relevant stakeholders from the communities that are shaping digital learning environments, to continue to develop this field of study.

**Table 1.** Digital Learning Design Principles Informed by Digital Learning Theory

Observational learning	Human learning occurs through observation. Within digital learning environments, individuals could engage in object-mediated learning, like videos or avatars engage in behaviors are observed, and individuals could experience vicarious learning, which could be based on incentives for increasing positive behaviors such as prosociality or reducing risky behaviors through consequences.^81–83^
Positive reinforcement	Digital learning environments could be designed with immediate feedback, positive reinforcement, and extrinsic motivations for desirable behaviors.^40,84^ Enhancing learning, engagement, increasing positive affect, which in turn can foster positive virtual environments.^64,85^
Trauma-informed design	Designing environments that incorporate trauma-informed design principles (safety, trust and transparency, peer support, collaboration, empowerment, identity/generational impacts), these principles when implemented properly and moderated closely can provide an informed digital learning environment that increases psychological safety, delivers supportive services, and encourages individual control.^86–89^
Virtual support services	Digital learning environments can incorporate virtual support services for anyone struggling with mental health issues. These include counseling or mental health services that support individuals.^87,90,91^
Scaffolding digital learning environments	Scaffolding was pioneered by Vygotsky^92^ (and means providing assistance through engagement. Digital learning environments can provide resources such as tutorials, engaging agents, and guidance, which help individuals learn at their own pace; they can be paired with rewards as a means of encouraging exploration through safety.^93,94^

These constructs have been explored and researched in various areas related to technology use. References for each construct can be found in Refs.^40,79–83,84–92^

### Make technology less extractive

As a means of supporting human development, technology should be designed in a manner that makes it less extractive. Shoshana Zuboff has demonstrated that, currently, all digital platforms are designed in terms of requiring large amounts of user information or generating excessive data usage, which is then aggregated and used with the intention of understanding user behavior and persuading users to spend as much time as possible engaging with their platforms.^68^ Further research exploring less extractive technology patterns/motivations could encourage environmental exploration and interaction while respecting the privacy of their users and integrating financial dynamics that, at a minimum, can be clearly articulated to users, providing them with agency over their own data usage and potentially considering sharing incentives with users as a means of shared transparency for all parties.

### Implement robust safeguards

Crawford et al. report that the design of digital interfaces and protective tools can provide safeguards across age cohorts and improve outcomes, enhancing agency and psychological safety across generational cohorts.^69^ By providing better practices in security and privacy controls on devices, applications, and artificial intelligence, along with a commitment by platforms to implement a balance between antisurveillance measures and safe, reasonable moderation over content disseminated in these spaces, users can begin to experience safety in digital environments.

Rosen et al. demonstrated that appropriate levels of protection should be considered depending on the level of developmental maturity and understanding.^70^ These safeguards could include control over the types of users with which individuals can interact, including similar age cohorts. In addition, researchers such as Pennycook and Rand and Lewandowsky and team, among others, demonstrated that safeguards such as offering clear transparency about whom users are interacting with (e.g., types of users, bots) and whether information that is being shared is authentic and verifiable are essential to certify trust within these environments and support positive outcomes.^71–75^

### Promote positive psychology interventions

Digital learning environments can be designed with positive psychology interventions that can facilitate experiences such as positive resonance, emotional savoring, social support, prosocial behavior, self-transcendence, compassion, belonging, and more.^42,76,77^ There are several opportunities to explore and use technology for the good that are being investigated^45,78^ and understanding positive technology use is instrumental in the process of designing positive digital learning environments.

### Promote flourishing

Dahl et al. propose that the development of awareness, connection, insight, and purpose is an essential component of psychological and overall well-being.^20^ They explored the manner in which intentional mental training can be harnessed to enhance and support individual and collective well-being by developing self-regulatory processes as skill sets. Digital learning environments that center spaces for mental training exercises that are developmentally appropriate and culturally celebratory might then become spaces in which well-being is learned.

Drawing from trauma-informed, social, positive, and developmental psychological concepts, we have curated the additional digital learning design principles listed in Table 1, which we believe can help support the development of positive digital learning environments. These recommendations are based on a combination of established theories and empirical studies to help inform design in a way that makes digital learning environments more accessible, supportive, positive, and successful. They are meant to scaffold future conversations, intervention development, research, and design efforts (User Experience/User Interface) in the area of Digital Learning Theory, a field that we propose to delineate further in more detail in a future article.

## Digital Social Learning and Future Directions

Social Learning Theory's model of a person's behavior and environment provides context into what is occurring in the digital world. The gradual behavioral changes experienced when people interact online are the centerpiece of learning theory. The additional socialization process through digital learning environments shapes development through gradual cognitive organization changes. Currently, the digital learning environments people engage in may be creating a false sense of authenticity and an illusion of freedom in that environment.^79,80^ Rather than selecting parts of an environment that people feel they can activate, technology algorithms rapidly impose the most effective strategies for their engagement. In addition, when we consider the degree to which people may experience negative experiences in both the physical world and the virtual world (hate speech, racism, traumatic events, cyberbullying, and more), few areas in the virtual world offer a refuge for people to engage in healthy coping behaviors.

Many questions remain to be explored within the field of Digital Learning Theory. What modes are people using to engage in positive behaviors in these environments? How is people's understanding of their intersectional identity being shifted by their interactions in digital learning environments, and how much of this can be attributed to the function of algorithms in these spaces? How can digital learning environments be created to encourage the development of prosocial behaviors and positive emotions such as belonging, compassion, and empathy? Are some strategies more effective in virtual environments for increasing feelings of connection or exploration?

Furthermore, what do we know about how ACES are being mediated by the use of digital learning environments? Are there specific types of experiences and models that can counter the negativity bias of interactions? What principles from the Social Learning Theory are most helpful in creating positive behaviors in virtual environments? Are there well-being interventions (emotional, psychological, or physiological) that exist in the disciplinary fields of education, public health, or medicine that could inform the design principles behind the creation of human digital learning environments to support user well-being? These are some of the questions that can be further explored when applying social learning in digital learning environments. We believe that Social Learning Theory itself provides the very foundation that reflects a need to further articulate the nuances of Digital Learning Theory and explicitly define Digital Learning Theory as a field that should be further intensively developed.

## Conclusion

Digital Learning Theory demonstrates that reciprocal determinism exists as a constant interactive relationship between personal factors, behaviors, and environments where all elements are continuously influencing each other in digital environments such as metaverses, video games, and social media platforms. The manner in which digital environments have currently been shaped to manipulate learning opportunities, and by extension perceptions of self-agency, has demonstrated devastating effects on people (e.g., online aggression, cyberbullying, body shaming, comparison). The importance of creating a flourishing and productive environment for the success of learners was highlighted by social learning theorists decades ago. We have argued that learning is something that occurs across the life span, and therefore, it is imperative to consider that social learning is widely applicable to how we understand how humans develop their sense of self and their understanding of their role in digital worlds.

Furthering our understanding of the relationship between learning, the development of sense of self, identity, and the expression of digital agency, which occur in the context of reciprocal determinism, or the interactive relationship between personal factors, behaviors, and environments, can now be accomplished through the exploration of Digital Learning Theory. Even though positive information is readily available, the lack of conversation between the designers of digital learning environments and the scientists who study the psychology of well-being, flouring, and the cultivation of positive emotions and relationships means that the inclusion of fundamental constructs such as vicarious reinforcement, model environments, self-efficacy, the power of cultural belief systems, self-agency, motivational and production processes, and more, is not happening in meaningful ways. This reflects a significant gap in the technology development and psychological processes. This gap demonstrates opportunities that can be explored to consider the “users” in the design of a humane metaverse.

Through Social Learning Theory, we can see how a digital learning environment can contribute to the development of trauma-informed digital spaces that prioritize the needs of the user. When using social learning and positive psychology theories in the development of positive technology experiences, these tools can lead to beneficial outcomes such as positive interactions and connections with peers while building adaptive capacities, self-efficacy, and resilience, which are particularly important when people experience personal obstacles or may be struggling through isolation or global pandemics such as COVID-19. As mentioned in Table 1, digital learning environments can also be utilized to connect people with valuable digital mental health resources that are personalized to their particular experiences and are supplemental to those available in the real world.

When used as a positive tool, positive social learning environments encourage exploration, novelty, and connection. Technology built with the tenets of positive psychology at its core can cultivate healthy development and personal and community-based resources by improving prosociality, self-regulation, positive behaviors, and learning through access to safe digital spaces. More importantly, social learning can provide insights into how researchers, designers, policymakers, educators, and parents can help facilitate healthy human development and support intervention development. Many of these concepts are not fully understood by engineers and programmers who have designed them. However, by encouraging technologists to work more openly with researchers in positive psychology and well-being scientists, technology design can become better informed as a tool for positive social and emotional change. Focusing on outcomes through a positive developmental framework through building positive digital learning environments can better shape a humane metaverse as well as other digital domains.

These efforts can inform the implementation of the best strategies for well-being in these spaces and shape behaviors and environments that enhance our ability to use technology in beneficial ways. Our species and the planet depend on it.
